# Supplementary material for: Venomix: a simple bioinformatic pipeline for identifying and characterizing toxin gene candidates from transcriptomic data
Source: PeerJ. 2018 Jul 31;6:e5361. doi: 10.7717/peerj.5361 (PMC6074769; doi:10.7717/peerj.5361)
Supplement: Supplemental Information 3 [file peerj-06-5361-s003.gz › FinalOutput_E-6/Acetylcholinesterase-1_1/finaltree.pdf]

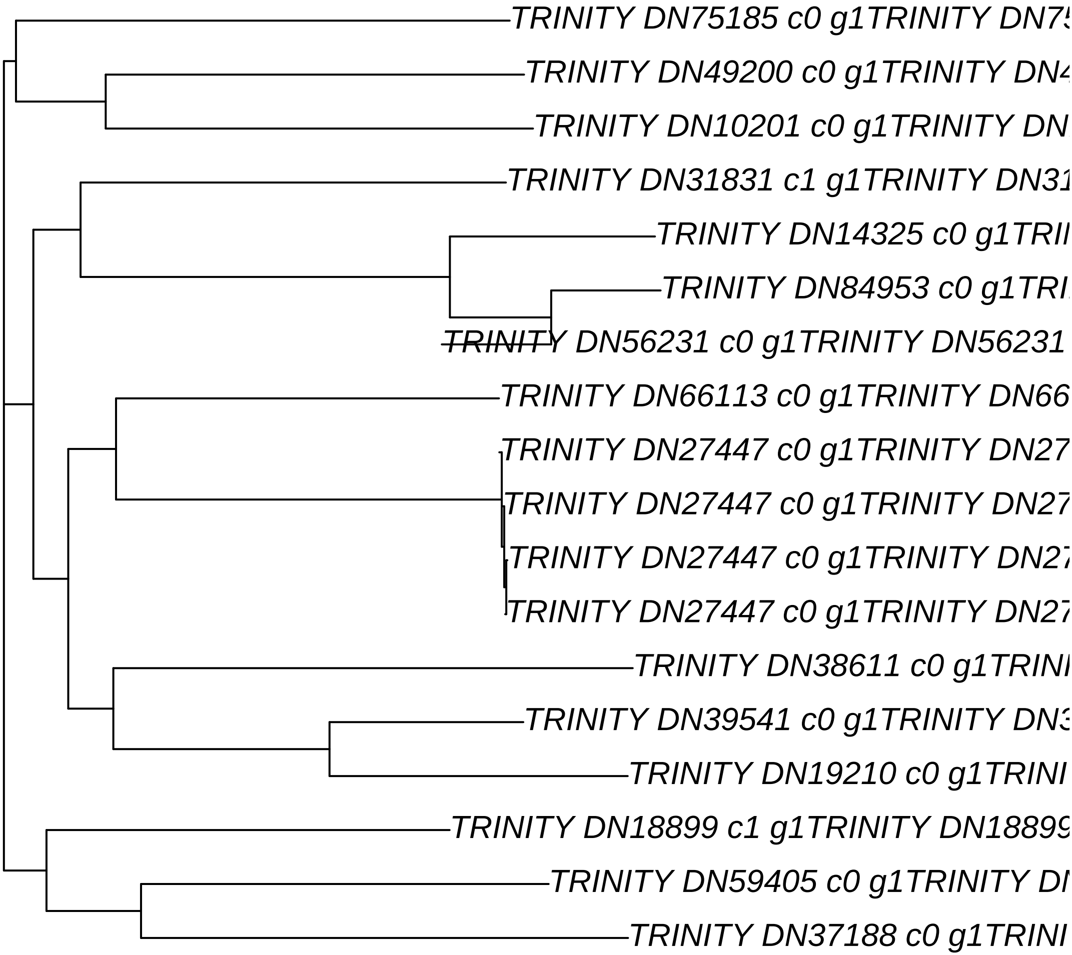
TRINITY DN75185 c0 g1TRINITY DN75
TRINITY DN49200 c0 g1TRINITY DN4
TRINITY DN10201 c0 g1TRINITY DN
TRINITY DN31831 c1 g1TRINITY DN31
TRINITY DN14325 c0 g1TRII
TRINITY DN84953 c0 g1TRI
TRINITY DN56231 c0 g1TRINITY DN56231
TRINITY DN66113 c0 g1TRINITY DN66
TRINITY DN27447 c0 g1TRINITY DN27
TRINITY DN27447 c0 g1TRINITY DN27
TRINITY DN27447 c0 g1TRINITY DN27
TRINITY DN27447 c0 g1TRINITY DN27
TRINITY DN38611 c0 g1TRINI
TRINITY DN39541 c0 g1TRINITY DN3
TRINITY DN19210 c0 g1TRINI
TRINITY DN18899 c1 g1TRINITY DN1889
TRINITY DN59405 c0 g1TRINITY DI
TRINITY DN37188 c0 g1TRINI
